# Disrupting the browsing experience: impact of sponsored social media content on affective flow without driving engagement

**DOI:** 10.3389/fnins.2025.1636848

**Published:** 2025-09-02

**Authors:** Maike Hübner, Julia Thalmann, Jörg Henseler

**Affiliations:** ^1^Usability and Interaction (UnI) Laboratory, Department of Business Administration and Economics, Ruhr West University of Applied Sciences, Mülheim, Germany; ^2^Department of Design, Production and Management, University of Twente, Enschede, Netherlands; ^3^Nova Information Management School, Universidade Nova de Lisboa, Lisbon, Portugal

**Keywords:** affective processing, social media advertising, emotional rhythms, facial expression analysis, galvanic skin response, user engagement

## Abstract

**Introduction:**

Understanding how emotional experiences shape consumer behavior in digital environments is a central issue in decision-making neuroscience. While social media feeds are saturated with sponsored content, little is known about how such content modulates affective rhythms and influences engagement.

**Methods:**

Grounded in decision neuroscience frameworks and affective processing models, this study develops a three-layer analytical model to capture the emotional microstructure of scrolling behavior, conceptualized as the micro-customer journey. Participants navigate a simulated social media feed while responses were recorded via facial expression analysis, skin conductance, and real-time engagement tracking.

**Results:**

Browsing was predominantly neutral in affective tone, interrupted by brief spikes in arousal and positive valence. Sponsored content disrupted this baseline neutrality, producing a subtle shift in affective flow without amplifying emotional intensity. Contrary to common assumptions, biometric indicators of emotional arousal and valence did not predict engagement behavior.

**Discussion:**

Findings suggest that commercial content influences decision-making not by heightening emotional salience but by interrupting habitual affective continuity. This challenges conventional persuasion models that emphasize emotional intensity and highlights the need for revised frameworks that account for rhythm disruption, cognitive reappraisal, and trait-level variability in user responses.

## 1 Introduction

In contemporary consumer research, the concept of the customer journey serves as a foundational framework for analyzing how individuals interact with brands across multiple stages and touchpoints over time. Typically segmented into pre-awareness, consideration, purchase, post-purchase, and loyalty phases (Court and McKinsey and Company, 2009; Lemon and Verhoef, 2016), each stage involves distinct emotional, cognitive, and behavioral processes that influence consumer decision-making and long-term brand relationships (Becker et al., 2020; Jaakkola and Alexander, 2024).

Within this framework, early-stage interactions, especially during pre-awareness and consideration, are critical. Studies have shown that the initial attention and affective impressions formed in these phases significantly shape downstream outcomes, including brand recall, evaluation, and conversion likelihood (Azzari and Pelissari, 2020; Gerou, 2022). However, despite their strategic importance, these phases remain understudied in terms of moment-to-moment affective processing, particularly in digital browsing. Therefore, this study focuses on the browsing experience, which primarily corresponds to the pre-awareness and consideration phases, as these are the most commonly utilized touchpoints for increasing user interest, and often constitute the first point of contact between users and brands (here ads) (Lemon and Verhoef, 2016; Guo et al., 2024).

Current knowledge of digital advertising, especially native advertisements seamlessly embedded in social media feeds (Johnson et al., 2019; Kim et al., 2019; Aribarg and Schwartz, 2020), centers on their effects on recall, persuasion, and advertisement recognition (Apostol, 2020; Pierre, 2024). Although effective in these terms, native advertisements introduce unique attentional and emotional dynamics: they are designed to blend in; however, their persuasive intent can subtly interrupt or hijack the emotional flow of users. Prior studies have rarely captured how these microevents are experienced in the moment or how they influence behavior, relying instead on retrospective self-reports that are vulnerable to bias (Brown et al., 2017).

Emerging work in cognitive neuroscience and attention research suggests that brief advertising encounters may have deeper consequences than previously assumed. For example, brief stimuli can influence working memory encoding, attentional prioritization, and emotional salience (Turk-Browne et al., 2013; Salahub et al., 2019). Moreover, advertisements that violate feed expectations may provoke attentional disruption (Uncapher et al., 2011), thereby interfering with affective continuity and downstream decision-making. Collectively, these findings highlight the need to explore how advertisements interact with the emotional states of users in real time, and not just after this fact.

To address this critical gap, this study aimed to investigate how users emotionally process and behaviorally engage with marketing content embedded within social media feeds. Targeting the pre-attentive and early consideration stages of the customer journey, and focusing on in-the-moment emotional and behavioral responses during feed browsing, the following research questions emerge:

RQ1 – Affective baseline: What is the emotional rhythm of social media browsing?

RQ2 – Post type effects: Do affective responses vary as a function of post type (sponsored vs. organic)?

RQ3 – Affect–behavior link: How do momentary affective states influence engagement behavior?

To answer these questions, we conceptualize social media feeding as a micro-customer journey–a sequence of dynamic and emotionally variable interactions nested within broader stages of the customer experience. Within this framework, in-feed native advertisements are treated not as isolated stimuli but as embedded affective events, whose impact depends on their interaction with the emotional and attentional flow of the surrounding content. To investigate these dynamics, we adopted a biometric approach using Galvanic Skin Response (GSR) and Facial Expression Analysis (FEA) to capture real-time physiological indicators of arousal and valence as users navigate a simulated Instagram feed. This layered structure, applied within a cohesive experimental design, is novel in the literature and moves beyond *post hoc* inference toward a richer understanding of the neuroaffective underpinnings of digital engagement.

## 2 Materials and methods

### 2.1 Theoretical framework and conceptual model

Building on the research questions outlined in the Introduction, this section lays the theoretical foundation for this study by integrating classical consumer behavior models with contemporary theories of attention, affect, and digital interaction. Specifically, this study draws on the Stimulus–Organism–Response (S–O–R) framework, the Cognition–Affect–Behavior (CAB) sequence, and predictive coding to conceptualize how consumers emotionally experience embedded advertisements during social media scrolling. Subsequently, these frameworks were translated into a layered analytical model that guided our empirical investigation.

The S–O–R model (Mehrabian and Russell, 1974) has long served as a foundational framework for understanding consumer reactions to environmental stimuli. In this model, a stimulus (S) elicits internal affective or cognitive states within the organism (O), which triggers a behavioral response (R). The model emphasizes the mediating role of emotional and cognitive processes, positing that consumer behavior is not a direct function of exposure but rather of internal interpretation and emotional appraisal. In this study, the model provides a processual lens for understanding emotional experiences as they unfold during digital interactions. We define S as the type of content encountered, specifically, whether a post is sponsored or not. O refers to the affective state of the user, operationalized through biometric measures of arousal (via GSR) and valence (via FEA). R is defined as observable behavioral engagement, measured by whether a user likes a given post.

The CAB model (Lavidge and Steiner, 1961; Holbrook and Batra, 1987) outlines a sequential flow in consumer decision-making, wherein cognitive evaluation precedes emotional reactions, which in turn inform behavioral intent or action. Although traditionally applied to advertising and persuasion contexts, the CAB model has also been adapted to describe subconscious or automatic affective responses, especially in environments with low cognitive elaboration, such as social media (Coles and Saleem, 2021). Both models converge on the principle that affective states act as critical intermediaries between marketing stimuli and consumer actions. This theoretical bridge is particularly relevant in digital contexts, where users are often exposed to rapid sequences of content and make behavioral decisions (such as clicking, liking, and sharing) with minimal conscious deliberation.

Although S–O–R and CAB remain relevant, their application in digital and mobile environments requires adaptation. Unlike traditional advertising, which is often deliberate and temporally discrete, social media feeds present continuous, immersive, and fast-paced streams of content. Here, users scroll quickly through mixed-source posts, including personal updates, influencer content, and in-feed brand placement. Therefore, they engage in low-involvement, habitual consumption of information (Meier et al., 2023; Piya and Adhikari, 2023). In such contexts, consumer reactions are shaped less by rational evaluations and more by pre-reflective, emotionally charged responses. Drawing on dual-process theories (Kahneman, 2011), this digital context activates System 1 processing, which is fast, automatic, and emotionally driven. Consequently, consumers may not consciously evaluate the persuasive intent of an advertisement but still experience affective disruption or congruence as it unfolds within the stream. This underlines the emotionally charged nature of the browsing experience and highlights the need for a clearer understanding of its affective load regarding arousal, as well as the distribution of valence: positive, negative, or neutral.

To capture this reality, we propose the concept of the micro-customer journey. A bounded segment of interaction within the broader customer journey, characterized by moment-to-moment emotional fluctuations and ephemeral engagements. Although broader journey models track longitudinal changes across the pre-purchase, purchase, and post-purchase phases (Court and McKinsey and Company, 2009; Lemon and Verhoef, 2016), the microjourney framework focuses on intra-experiential dynamics, particularly how individual posts and embedded advertisements modulate affective flow. Here, we incorporated predictive coding (de-Wit et al., 2010), which posits that the brain continuously generates expectations about incoming stimuli and updates these predictions based on sensory input. In a social feed context, organic posts may typically align with user expectations and maintain emotional continuity. However, even when visually native, sponsored posts may introduce affective prediction errors that disrupt attentional focus or emotional momentum. This underscores the need for a deeper understanding of the effect of post type (sponsored vs. organic), particularly discussing its potential impact on disrupting the affective flow of the browsing experience as part of the micro-customer journey.

However, these disruptions do not necessarily need to be negative but may be informationally salient, potentially triggering physiological arousal and facial affective responses. Thus, we treat social media feeds as a temporally affective environment where affectively incongruent content (such as native advertisements with persuasive cues) can create micro-disruptions in the emotional trajectory of the user. These disruptions may also inform subsequent engagement decisions, such as whether to like a post. This raises the question of whether users are more likely to like a post that evokes above-average emotional intensity, and whether this affect–engagement relationship is moderated by post type.

To empirically investigate these dynamics, we structured our study around a three-layer analytical framework, each informed by the theories outlined above. This layered model enables us to systematically assess the ambient affective experience of browsing (Layer 1), impact of sponsored versus organic content on affective responses (Layer 2), and predictive link between emotional salience and behavioral engagement (Layer 3). Each layer corresponds to a component of the S–O–R and CAB models and is operationalized using biometric and behavioral data. Table 1 summarizes this alignment.

**TABLE 1:** Conceptual alignment of analytical layers with the S–O–R and CAB frameworks.

Layer	Theoretical component	Psychological process	Measurement	Research focus
Layer 1	Organism (baseline)	Ambient affective state	GSR (arousal), FEA (valence)	What is the emotional rhythm of social media browsing?
Layer 2	Stimulus → organism	Post-type affect modulation	GSR and FEA × post type	How do native ads disrupt or align with emotional flow?
Layer 3	Organism → response	Affect-driven behavior	GSR/FEA deviations, Likes	Do affective spikes predict engagement behavior (likes)?

S–O–R = Stimulus–Organism–Response; CAB = Cognition–Affect–Behavior; FEA = Facial Expression Analysis; GSR = Galvanic Skin Response.

This layered model allows us to bridge the macro-level theory (S–O–R, CAB) with micro-level measurements in a naturalistic, platform-native context.

To implement this model, three key constructs were measured: (1) Arousal, captured via GSR, reflects sympathetic nervous system activity and serves as a well-established index of emotional intensity (Sharma and Nayak, 2019; Shehu et al., 2023), (2) Valence, inferred through FEA, is assessed using a validated computer vision system that classifies frame-by-frame facial emotion expressions (Ekman and Friesen, 1976; Stöckli et al., 2018), (3) Behavioral engagement, operationalized as “liking” a post (Schreiner et al., 2021), represents the most immediate and low-cognitive-effort form of user interaction in social media.

These complementary measures capture both implicit emotional responses and explicit behavioral outcomes, allowing for a high-resolution analysis of how emotional fluctuations within a feed shape momentary decision. Building on the three-layered analytical framework described above, we derive a set of testable hypotheses that align with each stage of the emotional and behavioral dynamics theorized in digital scrolling contexts. The first layer focuses on laying the foundation for understanding the underlying advertising experience in which advertisements are embedded. The design of social media platforms, particularly the feed-based architecture of applications such as Instagram, has increasingly come under scrutiny for its psychological and emotional impact. Although designed to enhance connectivity and content discovery, feeds are experienced not only as information channels but also as affective environments. Continuous exposure to social comparison, curated success, and endless novelty fosters emotional instability, particularly among young users (Tackett et al., 2014; Muthuraman, 2024). The Fear of Missing Out, need for social validation, and highlight-reel distortion of the lives of others result in feelings of inadequacy, envy, anxiety, and even depressive symptoms (Piteo and Ward, 2020; Du et al., 2024). Therefore, the layer sheds light on the emotional stress of such a browsing experience. Based on previous studies on affective rhythm (Martínez-Guzmán and Lara, 2019; Breek et al., 2021) and attentional filtering (Tremblay et al., 2015; Li and Deng, 2024), we propose the following research propositions that inform Layer 1 of our framework:

RP_1*a*_: The browsing experience is characterized by moderate phasic arousal, with variability across posts.
RP_1*b*_: The browsing experience is primarily neutral in valence, punctuated by occasional positive or negative expressions.

These propositions serve to describe the affective baseline from which content-specific modulations (Layer 2) can be interpreted. They reflect emergent emotional structure rather than predictions tested through inferential falsification.

The second layer introduces stimulus-level variance by investigating whether native advertisements, despite being visually integrated, elicit different affective responses to organic content. This layer enhances the overall understanding of the already saturated emotional context by focusing on post type (sponsored vs. organic). Sponsored posts, also referred to as native advertising (NA), are commercial content seamlessly integrated into organic social feeds, potentially disrupting the browsing experience. By nature, NA blends in using platform-congruent visuals, language, and placement to reduce user resistance and enhance engagement (Campbell and Marks, 2015; Wojdynski and Evans, 2016). However, based on predictive coding and the literature on advertisement recognition and resistance (Bakalash and Riemer, 2013; Lin and Kim, 2016; Akgun et al., 2017), we formulate the following research propositions to guide our biometric comparison of content types:

RP_2*a*_: Sponsored posts are characterized by higher phasic arousal than organic posts.
RP_2*b*_: Sponsored posts are characterized by lower valence (that is, fewer positive expressions) than organic posts.
RP_2*c*_: Organic posts are characterized by more neutral expressions than sponsored posts.

These propositions aim to assess whether emotionally incongruent or commercially salient stimuli disrupt the affective continuity of scrolling. As such, they offer a descriptive framework for understanding how native advertising may operate as a micro-disruption within an otherwise ambient and emotionally neutral feed experience.

The third layer links the internal affective states to observable behaviors. Consistent with the CAB model and dual-process theories of digital decision making (Lavidge and Steiner, 1961; Kahneman, 2011), we investigated whether emotionally salient moments predict user engagement. Specifically, this layer examines whether moments when the arousal or valence of users significantly exceeds their average levels predict behavioral engagement. We define action as the act of liking a post, and test whether affective salience increases the probability of engagement. This layer also examines whether this relationship is moderated by post type, that is, whether users are more likely to act on emotional responses to organic content. Therefore, we formulate the following research propositions, designed to guide our inferential investigation into how emotional salience may, or may not, translate into action:

RP_3*a*_: Posts that are characterized by above-average arousal or valence are also characterized by a higher likelihood of receiving behavioral engagement (likes).
RP_3*b*_: The affect–engagement association is expected to be more pronounced for organic posts than for sponsored posts.

These propositions reflect theoretically informed expectations rather than confirmatory hypotheses. They aim to explore whether emotional micro-dynamics embedded in the browsing stream align with subsequent behavioral responses. Given the habitual and low-effort nature of feed interactions, affective resonance may not consistently translate into engagement. Therefore, this layer is designed to investigate possible affect–action coupling within a structurally volatile digital environment. The following section outlines the implementation of this layered analytical model in a controlled and ecologically valid experimental setting. We detailed the experimental design, stimulus development, data collection tools, and analytical strategies used to quantify biometric and behavioral data at the post-level. This approach establishes a robust empirical foundation for testing the hypotheses derived from the theoretical framework presented here.

### 2.2 Research design

Building on the conceptual framework introduced above, we operationalized the micro-customer journey of social media browsing as a temporally compressed sequence of post encounters punctuated by intermittent brand messages. To capture the rapid, affect-laden nature of this journey, we implemented a within-subjects laboratory experiment in which each participant scrolled through a simulated Instagram feed while continuously monitoring their psychophysiological state. A within-subject design was chosen because it provides two methodological advantages pivotal to this study. First, using each participant as their control minimizes inter-individual variability, which is an important consideration when emotional reactivity varies widely among users (Wickens and Keppel, 2004). Second, by exposing each participant to both sponsored and organic posts, we created robust within-person contrasts that mirrored the way users encounter mixed content in live feeds.

To preserve ecological validity, the interface emulated the familiar mobile form factor: participants scrolled vertically through 29 posts (8 sponsored, 21 organic) presented in a random order but with fixed dimensions. This 1:3 advertisement-to-content ratio reflects typical Instagram densities (≈20%–25%) (WhistleOut, 2022) yet remains conservative relative to peak exposures (up to 42%). Participants could browse at their own pace for up to 150 s, a ceiling that approximates the mean session length of 164 s reported for Instagram (Soax, 2024) while still ensuring sufficient time for reliable biometric capture. By avoiding a forced-exposure paradigm in which stimuli are displayed for a predetermined interval, we respect the fast-scroll behavior characteristic of contemporary feed consumption (Windels et al., 2018; de Keyzer et al., 2023).

The stimulus set was curated to balance experimental control with real-world relevance. Sponsored posts were collected from active advertising campaigns spanning fashion, travel, food, media, music, and fitness niches (Heepsy, 2023). All included the platform-mandated “Sponsored” disclosure, thereby preserving regulatory authenticity. Organic posts, in turn, were sourced from private accounts but stripped of personal identifying markers to eliminate familiarity bias. To mitigate order and fatigue effects (Miller, 2005; John and Révész, 2020), a full pool of 29 posts was reshuffled for three created feeds, which were randomly assigned to participants. This ensured that any single advertisement was equally likely to appear early or late in the browsing sequence. This study was approved by the ethics review board of the university of Twente (protocol number: 230564).

### 2.3 Data collection: physiological measurements

**FIGURE 1:**
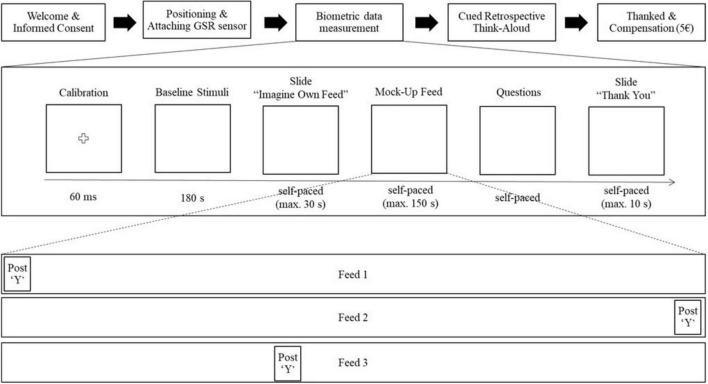
Experimental procedure and mock-up feed structure.

Data collection was conducted in a controlled laboratory environment using iMotions 9.3 for synchronized biometric data acquisition. Sessions (∼30 min) followed a standardized procedure (illustrated in Figure 1), beginning with informed consent, sensor calibration, and a 3-min baseline. Participants then completed a self-paced browsing task within a simulated Instagram environment, followed by a retrospective think-aloud task. GSR and FEA were recorded continuously to ensure rich, temporally aligned biometric datasets. However, retrospective think-aloud data as well as further biometric data (e.g., eye-tracking) were collected and part of a separate study (Hübner et al., 2025).

#### 2.3.1 GSR: procedure and metrics

In this study, the GSR was recorded continuously throughout the browsing session using the Shimmer3 device, with a frequency range of DC–15.9 Hz. Electrodes were attached to the palmar surfaces of the non-dominant hand, following standardized psychophysiological procedures (Dawson et al., 2007). Given the continuous, self-paced nature of the browsing task, participants encountered posts at variable durations and in a naturalistic sequence. Classical event-related averaging based on fixed stimulus onsets was therefore unsuitable. Instead, we employed a post-level, event-based analysis in which each post was treated as a discrete annotated event. GSR features were extracted from the time window corresponding to each participant’s dwell time on that post, allowing arousal responses to be linked directly to specific content exposures. Signal processing was conducted using iMotions’ implementation of the peak detection algorithm. This procedure extracts the phasic component via median filtering (phasic filter length = 4000 ms), applies a low-pass Butterworth filter (cutoff = 5 Hz), and detects peaks based on onset threshold (0.01 μS), offset threshold (0.005 μS), amplitude threshold (0.005 μS), and minimum duration (300 ms). Gaps shorter than 4000 ms were linearly interpolated, and the option to remove discontinuities caused by sensor range switching was disabled. These parameter choices are consistent with recommendations in the psychophysiology literature (Dawson et al., 2007) and tuned to capture rapid, transient responses in a fast-paced, naturalistic setting. Two metrics were extracted for analysis:

1. Phasic Peaks: A binary variable indicating whether at least one GSR occurred during the post exposure (0 = no peak; 1 = ≥1 peak);
2. Average Peak Amplitude: The mean amplitude (in μS) of all detected peaks during exposure to a given post. This continuous measure reflects the intensity of physiological arousal and strength of the user’s sympathetic activation in response to the content.

This approach aligns with recent applied affective computing and UX research in which phasic peak features are widely used as primary indicators of sympathetic activation (e.g., Marques et al., 2024; Nhan et al., 2025; Mandić et al., 2023). As Lal et al. (2024) note, there is currently no consensus on whether tonic or phasic components are preferable (Horvers et al., 2021), and our focus on peak incidence and amplitude was chosen for their theoretical relevance to momentary arousal and empirical comparability. All data were visually inspected for signal quality, with segments affected by motion artifacts or technical dropout excluded from analysis.

**FIGURE 2:**
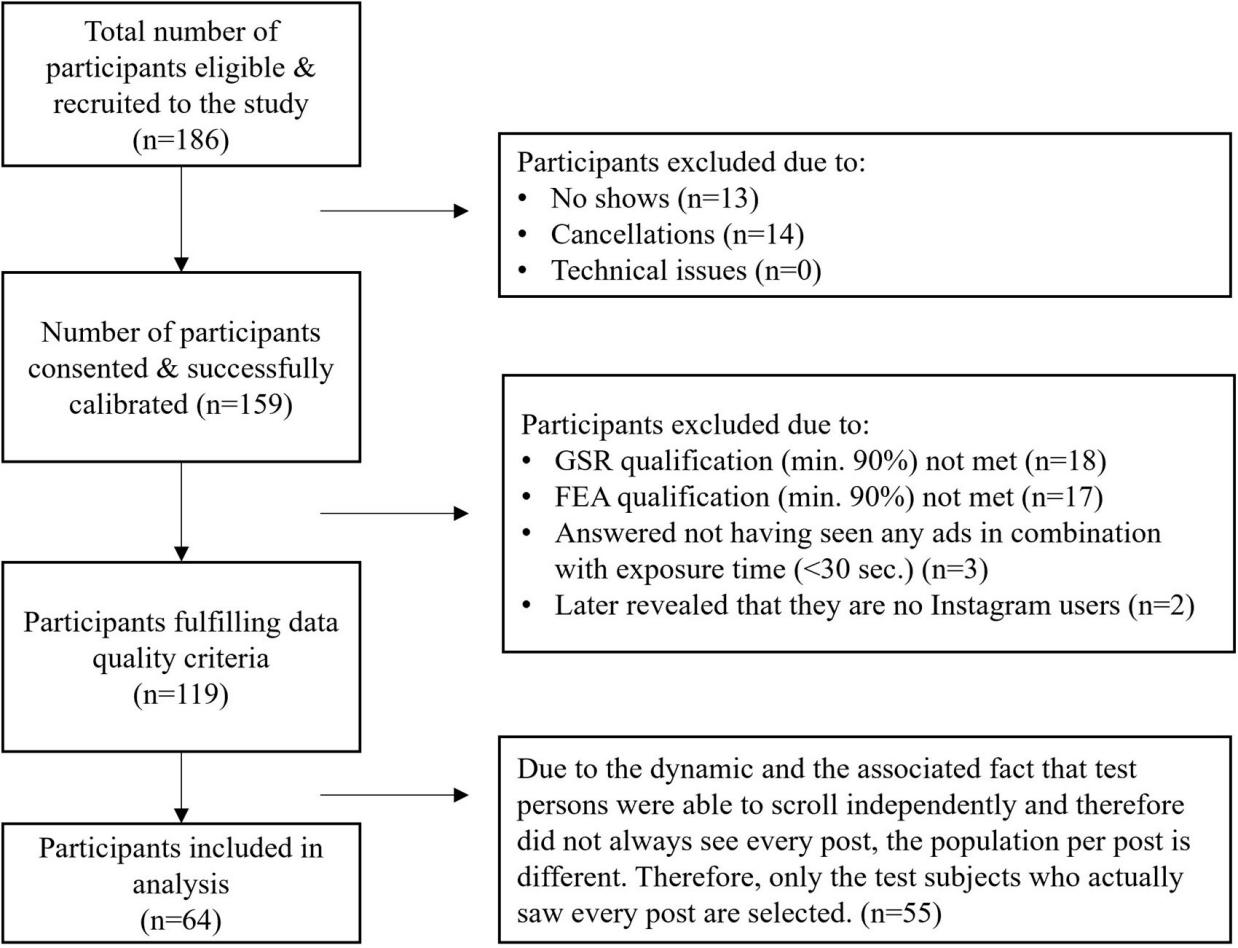
Participant flowchart detailing eligibility criteria for analysis.

Data quality was continuously monitored. Segments with sensor detachment, excessive movement artifacts, or calibration drift were identified through both automated flagging and manual inspection. Short gaps (<4000 ms) were interpolated; longer gaps were marked as missing. Participants with <90% valid GSR samples across the session were excluded from analysis (*n* = 18; Figure 2).

#### 2.3.2 FEA: procedure and metrics

Facial expressions of participants were recorded throughout the browsing session using iMotions Affectiva. Analysis was conducted using the following parameter settings: time bin length = 500 ms, no bin overlap, emotion channel threshold = 50%, action unit threshold = 50%, and valence threshold = 50%. These thresholds ensured that only facial expressions meeting a minimum classification confidence were included in the aggregated metrics.

Three FEA-derived metrics were used for analysis in this study: positive frames (such as smiling and eyebrow raises), negative frames (including frowning and brow furrows), and neutral frames (absence of emotional expressions). Specifically,

1. Positive Frames ≥ Threshold: The number of video frames classified as exhibiting a positive emotional expression above a pre-defined intensity threshold. This metric captures the prevalence of positive affect during content exposure.
2. Negative Frames ≤ Threshold: Analogous to positive frames but for negative emotional expressions. This metric indicates the prevalence of negative affect elicited by a given post.
3. Neutral Frames Between Threshold: The number of frames classified as neutral, representing emotionally non-valent or baseline expressions. This metric is crucial for distinguishing between emotional activation and neutrality during content processing.

By triangulating these three indicators, we mapped users’ emotional directionality as they navigated the feed, supplementing the arousal data obtained from the GSR with rich valence-specific information. This enables full circumplex positioning of each post interaction, capturing high or low arousal combined with positive, negative, or neutral affective valence. However, only facial tracking segments meeting minimum quality standards, such as including unobstructed key facial landmarks, adequate lighting, and stable frontal head orientation, were retained. Automated tracking confidence scores provided by AFFDEX were used to flag low-quality frames; those with confidence below 90% were marked as missing. Segments in which more than 50% of frames fell below this confidence threshold were excluded from analysis at the post level. Participants with fewer than 90% valid frames across the browsing session were excluded from the FEA analysis entirely (*n* = 17; Figure 2). This combination of strict inclusion thresholds and parameterized confidence filtering ensured that only reliable facial expression data contributed to the final analyses.

### 2.4 Participant recruitment and inclusion process

A total of 186 participants were recruited via convenience and snowball sampling within the university community and surrounding areas over a 3-weeks period (01–21 December 2023). Recruitment utilized digital channels (university websites and social media) and physical outreach (flyers and posters) to maximize diversity while facilitating laboratory data collection. After exclusion owing to the data quality of the GSR or FEA (min. 90%), or non-use of Instagram, 119 participants remained. Owing to the free browsing task design, not all participants were exposed to all posts, as some took longer to scroll through the mock-up feed than others. In line with our S–O–R framework and to ensure that emotional reactions could be meaningfully linked to exposure, only participants who saw every post were retained. This final filtering step resulted in a sample of 64 participants (51.6% female, 48.4% male, M_age = 24.73, SD = 5.40) (Figure 2), closely reflecting Instagram’s core user demographic, which skews toward adults aged 18–34 with an approximately balanced gender distribution (Statista, 2025).

## 3 Results

This section presents the results of our biometric and behavioral analyses, structured based on the three-layer analytical model derived from the S–O–R and CAB frameworks. Our analytic strategy was guided by hypotheses H1a–H3b and is organized accordingly. Layer 1 examines the affective rhythm of the feed as a baseline, which allows us to establish a foundational understanding of the affective baseline of the user. Layer 2 investigates the systematic modulation based on post type (sponsored vs. organic), thereby adding to Layer 1 by determining whether and how the baseline is modulated by commercial content. Layer 3 explored whether affective salience predicts engagement behavior.

### 3.1 Layer 1: baseline affective load in the browsing experience

As the first analytical layer in our model, Layer 1 captures the foundational emotional tone of the browsing experience, independent of post-type. Grounded in the S–O–R and CAB frameworks, this layer reflects the “Organism” component, where affective states emerge in response to the continuous stream of stimuli. Understanding this baseline is critical for interpreting how subsequent content-specific disruptions may modulate the user experience. To characterize the general affective dynamics of feed browsing, we first analyzed biometric patterns independent of content type.

**FIGURE 3:**
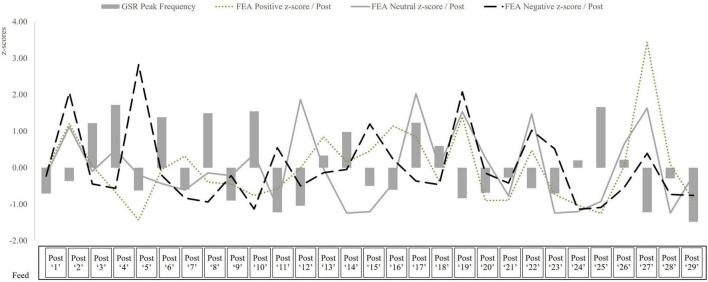
Affective dynamics across the scrolling feed.

Electrodermal activity, recorded via the GSR, revealed a browsing experience punctuated by episodic arousal peaks. The mean number of phasic skin conductance responses (peaks) per post was 10.59 (SD = 3.20), ranging from 5 to 18. The Z-scored data identified three posts as significantly above the mean (*z* > 1.96), indicating discrete episodes of elevated sympathetic arousal. The mean peak amplitude across all posts was 0.09 μS (SD = 0.0318). Although within normative bounds, one post approached significance (*z* = 1.72, *p* = 0.08). FEA revealed a feed experience dominated by neutral affect, punctuated by short-lived affective expressions. The mean number of neutral frames per post was 86.79 (SD = 26.94). Positive affect was moderate (*M* = 53.97, SD = 27.50), with one post generating a z-score of 3.44 (*p* < 0.001), indicating a strong, shared moment of emotional elevation. Negative affect was comparatively low (*M* = 31.54, SD = 25.40), and no post reached significance. One post approached significance (*z* = 2.03, *p* = 0.06). These data support hypotheses H_1*a*_ and H_1*b*_. Thus, understanding this baseline rhythm contextualizes the salience of specific stimuli, as explored in Layer 2. To visualize the fine-grained modulation of affective flow across the scrolling experience, we plotted z-standardized arousal and valence indicators per post (Figure 3).

### 3.2 Layer 2: emotional disruption by content type

Layer 2 assessed whether affective responses differed systematically by post type. This tests whether affective flow is modulated by the commercial nature of content, operationalized as native (sponsored) or organic posts. To address this, repeated-measures General Linear Modeling (GLM) using SPSS was employed, leveraging within-participant comparisons to assess the physiological and emotional distinctions between both post types. This approach controls for inter-individual variability in baseline arousal and affects sensitivity, making it well-suited for interrogating within-stream variability. Variables were derived at the post-level (that is, aggregated across each post), preserving the temporal granularity of Layer 1 while allowing us to detect systematic differences attributable to content type. Therefore, five dependent variables were analyzed independently: three from FEA (positive, negative, and neutral affect) and two from electrodermal activity analysis (peak frequency and amplitude).

The FEA GLM results showed differential emotional engagement according to post type (Table 2). Although the positive affect result was not statistically significant, it showed a marginal trend suggesting higher expressions of positive affect during organic posts than during sponsored ones (F (1,63) = 3.56, *p* = 0.064, η^2^ = 0.05). Consistent with this but contrary to expectations, no significant differences were observed in negative facial expressions across sponsored and organic posts (F (1,63) = 1.16, *p* = 0.285). Mean expressions were low overall, and neither post type approached the critical threshold for eliciting shared negative affect. This is supported by the significant divergence in affective ambiguity of the neutral affect measure with a robust and statistically significant difference (F (1,63) = 28.83, *p* < 0.001, η^2^ = 0.31). Organic posts elicited substantially more neutral frames (*M* = 89.12, SD = 3.38) than did sponsored ones (*M* = 68.08, SD = 3.86).

**TABLE 2:** Repeated measures GLM results for GSR and FEA metrics by post type.

Dependent variable	Post type	Mean	SE	F (1, df)	*P*-value	η^2^ (partial Eta^2^)
FEA (positive)	Sponsored	3.01	0.88	F (1,63) = 3.56	0.064	0.053
	Organic	5.15	1.88			
FEA (negative)	Sponsored	0.42	0.36	F (1,63) = 1.16	0.285	0.018
	Organic	0.79	0.70			
FEA (neutral)	Sponsored	68.08	3.86	F (1,63) = 28.83	<0.001	0.314
	Organic	89.12	3.38			
GSR (frequency)	Sponsored	0.15	0.02	F (1,63) = 2.50	0.119	0.038
	Organic	0.18	0.02			
GSR (amplitude)	Sponsored	0.081	0.019	F (1,31) = 0.42	0.526	0.013
	Organic	0.074	0.013			

GLM = Generalized Linear Model; FEA = Facial Expression Analysis; GSR = Galvanic Skin Response; SE = Standard Error.

Results from the GSR-based GLM support the physiological resonance of commercial applications. The number of phasic GSR peaks, which is an established proxy for momentary sympathetic arousal, was marginally higher for organic posts (*M* = 0.176, SE = 0.023) than for sponsored ones (*M* = 0.147, SE = 0.023), although this difference was not statistically significant (F (1,63) = 2.50, *p* = 0.119, η^2^ = 0.04). This trend is consistent with the general pattern observed in the FEA data: Organic posts are more capable of eliciting subtle affective and attentional modulation. The Mean amplitude of the GSR peaks did not differ significantly across post types (F (1,31) = 0.418, *p* = 0.523).

**FIGURE 4:**
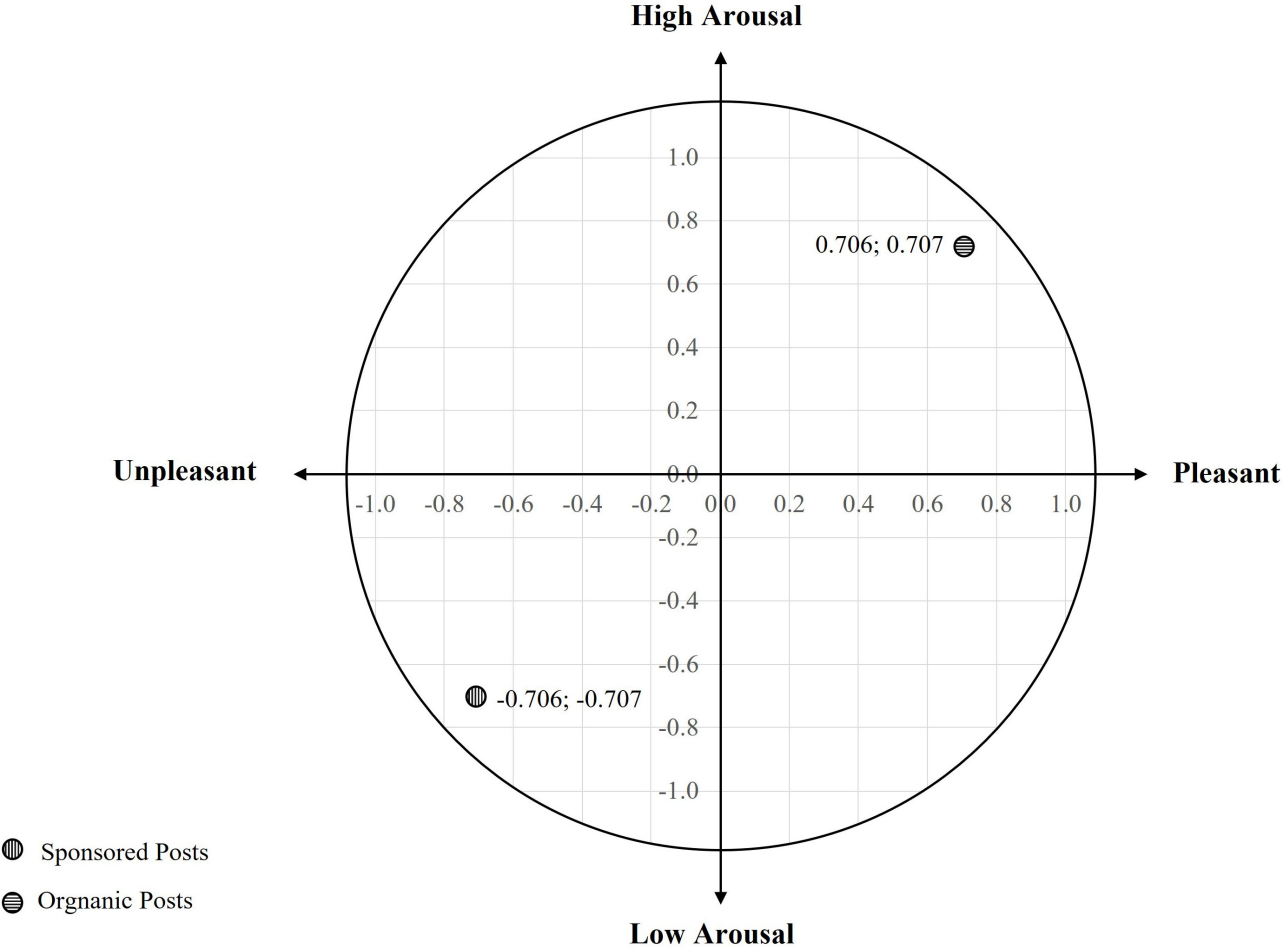
Circumplex model mapping of affective responses to sponsored vs. organic posts.

To conceptually synthesize these findings on biometric arousal and valence across content types, affective responses to sponsored and organic posts were mapped onto the Russell Circumplex Model of Affect (Russell, 1980) (illustrated in Figure 4). The z-score-based coordinate calculation is shown in Table 3. Russell’s (1980) model describes affective states along two fundamental dimensions: valence (pleasant–unpleasant) and arousal (high–low activation) (Russell, 1980). Although the Circumplex Model of Affect was originally conceptualized from self-reported affective states, a growing body of literature supports the approximate mapping of psychophysiological signals–particularly measures of autonomic arousal and facial expressions–onto its bidimensional structure (Pedersen, 2024). Importantly, our mapping approach does not claim a 1:1 overlap between physiology and subjective feeling; rather, we follow an accepted heuristic framework where biometric metrics are z-standardized and plotted as proxies for relative arousal (GSR) and relative valence (FEA) (Gong et al., 2024). This enabled comparative positioning within the affective space. Although our circumplex visualization remains a conceptual representation, it reflects both normative physiological associations and analytical conventions in the emotion research community.

**TABLE 3:** Z-score-based coordinate mapping of affective responses (valence and arousal) for sponsored vs. organic posts.

Measure	Condition	*M*	*M* of both	SD (pooled)	*X* coordinate	*Y* coordinate
FEA (valence)	Sponsored	3.014	4.083	1.513	−0.706	–
	Organic	5.152			+0.706	–
GSR (arousal)	Sponsored	0.147	0.1615	0.0205	–	−0.707
	Organic	0.176			–	+0.707

FEA = facial expression analysis; GSR = galvanic skin response; SD = standard deviation.

In conclusion, Layer 2 confirms that affective responses during social media browsing are not uniformly distributed across content types. Organic posts foster greater affective ambiguity (via neutral expressions) and exhibit trends toward increased positive affect and physiological engagement. In contrast, sponsored content appears less emotionally resonant and more structurally dissonant, breaking the flow without intensifying emotionality.

### 3.3 Layer 3: linking affective responses to behavioral engagement

Layer 3 addressed RQ3: “Do affective responses predict engagement behavior?” by investigating the predictive relationship between biometric indicators of arousal and valence (from GSR and FEA) and user behavior, operationalized as whether a user liked a post. This step marks advancement from describing affective resonance (Layer 1) and comparing structural emotional design (Layer 2) to explaining what drives user-operationalized as likes, a proxy for engagement. This operationalization was selected because it enabled unobtrusive, tap-based input on the dominant hand while GSR electrodes remained on the non-dominant hand and facial tracking required a stable head pose. More effortful behaviors, such as commenting, would have required head shifts and typing, introducing motion artifacts in the GSR signal and degrading FEA quality. Of the 64 users with complete data, the average number of liked posts was 2.9 of 29 (*M* = 2.86, SD = 2.95). Engagement was highly heterogeneous: 28% did not like any post, 38% liked one to three posts, and only 11% liked eight or more posts. The variance (8.69) exceeded the mean by a factor of three, indicating overdispersion. Therefore, we analyzed the counts using a negative binomial (NB) regression.

To test H3a, which posits that within-person fluctuations in emotional arousal predict the likelihood of liking a social media post, we first aggregated post-level liking responses into a user-level binary outcome (Liked_Any_Post), indicating whether a participant liked at least one post. This binary outcome was selected to align with the threshold models of affective decision-making and maintain robustness against outlier-driven skewness. Logistic regression provides optimal interpretability and statistical power for a given sample size (*n* = 64), enabling direct modeling of affect-to-behavior links. Second, we operationalized the behavioral outcome as an alternative to other count-based measures (such as total posts liked). This count-based alternative assesses cumulative engagement tendencies, rather than discrete decision-making moments. However, as the dependent variable showed class imbalance (that is, Liked_Any_Post, where ∼72% of users liked at least one post), we then decided to model at the post-level (multilevel) to increase the number of data points (*n* = posts, rather than users). Even though 72% of participants liked at least one post, they likely liked far fewer posts in total, resulting in a more balanced class distribution at the post-level.

Subsequently, we examined the variance structure of the data to determine whether a multilevel modeling approach was appropriate. Specifically, we estimated intercept-only (null) models for each post-level affective indicator to compute intraclass correlation coefficients (ICCs), which quantify the proportion of variance in the outcome attributable to between-person differences. Results from the null model for facial expression-derived arousal (FEA_Positive) revealed an ICC of.370, indicating that 37.0% of the total variance scores were due to differences among individuals, whereas the remaining 63.0% reflected within-person variance across posts. Similarly, for electrodermal arousal (GSR_Frequency), the ICC was.178, suggesting that 82.2% of the variance occurred at the within-person level. These results indicate substantial intra-individual variability in affective responses across posts. Given the hierarchical structure of the data (multiple posts nested within individuals) and sufficient within-person variation, the use of random-intercept multilevel logistic regression models was statistically and theoretically justified for hypothesis testing. To isolate within-person variability, each predictor was person-mean centered by subtracting individual means from each respective emotional response [FEA (positive), GSR (frequency)]. Descriptive statistics confirmed successful centering, with means of the transformed variables (*M* = 0.00), indicating that person-level baselines were effectively removed. This step allowed subsequent mixed-effects models to exclusively capture within-person fluctuations in emotional intensity and their predictive power for liking behavior. A random-intercept Bernoulli generalized linear mixed model (GLMM) (posts nested in participants; *n* = 1 785 after listwise deletion) showed no evidence that within-person deviations in electrodermal arousal (GSR_Frequency_C) or facial-expression arousal (FEA_Positive_C) increased the likelihood of pressing “Like,” (all|t| < 0.05, ps > 0.96). The marginal pseudo-R^2^ was < 0.001, indicating virtually no additional within-person explanatory power beyond the intercept. The random-intercept variance was σ^2^0^2^ = 27.49 (SE = 5.89, *p* < 0.001), yielding an ICC of.85, suggesting the most variability in liking resided between users rather than between posts (Table 4).

**TABLE 4:** Summary of models testing affective predictors of liking behavior (H3a).

Analysis	χ^2^/Wald-*z*	Effect size	95% CI	*P*
GLMM (post-level)	FEA64_C: *z* = 0.05	OR = 1.00	0.96–1.03	0.96
	GSR8_C: *z* = 0.01	OR = 1.00	0.14–7.20	0.99
	Marginal pseudo-R^2^ < 0.001; τ0^2^ = 27.49 (SE = 5.89), **ICC = 0.85**			
User-level logistic	Omnibus χ^2^ (2) = 1.49	–	–	0.48
	Mean_FEA64	OR = 1.02	0.96–1.09	0.57
	Mean_GSR8	OR = 6.72	0.22–210	0.28
Poisson vs. NB	Poisson Deviance/df = 3.31			
	NB Pearson χ^2^/df = 0.82	α≈1 (fixed)		
	Mean_FEA64	**IRR = 1.01**	0.98–1.03	0.49
	Mean_GSR8	**IRR = 1.08**	0.22–5.39	0.92

GLMM = Generalized Linear Mixed Model; ICC = Intraclass Correlation Coefficient; NB = Negative Binomial; IRR = Incidence Rate Ratio; CI = Confidence Interval; FEA = Facial Expression Analysis; GSR = Galvanic Skin Response; OR = Odds Ratio.

A Poisson model showed substantial over-dispersion (Deviance/df = 3.31; Pearson χ^2^/df = 3.13), indicating the variance in like counts far exceeded the mean. Switching to an NB model improved the fit [Akaike information criterion (AIC) = 347.3] (Table 5).

**TABLE 5:** Dispersion and information criteria for the poisson count model.

Metric	Value	Rule of thumb	Interpretation
Deviance/df	201.8/61 = 3.31	≈1 indicates a good Poisson fit	>1 ⇒ clear over-dispersion
Pearson χ^2^/df	191.2/61 = 3.13	Same rule	Confirms over-dispersion
AIC	347.3	Smaller is better (for model comparisons)	Baseline for later NB model
Log-likelihood	−170.6	Higher (less negative) is better	Will improve if NB fits better

AIC = Akaike Information Criterion; NB = Negative Binomial.

With a dispersion ratio > 3, the equi-dispersion assumption of the Poisson distribution is violated. Standard errors were underestimated, and *p*-values were overly liberal. Therefore, we used an NB specification for valid inference.

An NB GLM (log link) was used to test whether the average facial expression (Mean_FEA_Positive) and electrodermal (Mean_GSR_Frequency) arousals of the participants predicted the number of Instagram posts liked (*M* = 2.86, SD = 2.95; *n* = 64). Model fit was adequate (Pearson χ^2^/df = 0.82; Deviance/df = 1.15) and superior to a Poisson alternative (AIC = 288.1 vs. 347.3). However, neither predictor reached significance (incidence rate ratio [IRR] of Mean_FEA_Positive = 1.01, 95% confidence interval [CI][0.98, 1.03], *p* = 0.49; IRR of Mean_GSR_Frequency = 1.08, 95% CI[0.22, 5.39], *p* = 0.92). Thus, between-person differences in average arousal did not explain the number of posts users engaged in, echoing the post-level null finding.

Across all three specifications–momentary, binary, and count–arousal failed to predict liking. The GLMM showed that 85% of the variance in liking resides between users; adding within-person arousal increased explained variance by <0.1%. At the person level, higher average arousal did not distinguish engagers from non-engagers, nor did it explain the number of posts liked. Model fit indices confirmed that the NB family was appropriate; however, the affective covariates remained non-significant. The conclusion for H3a is that the hypothesis was not supported; biometric arousal, whether transient or averaged, does not translate into low-threshold engagement, such as pressing “like.” A zero-inflated NB model (log link) was estimated using *pscl* in SPSS software (v29, R-Essentials). Convergence was achieved (46 iterations, BFGS). Model fit (AIC = 291.7) was not superior to a standard NB model (AIC = 288.1; ΔAIC = 3.6). Neither mean facial-expression (Count β = 0.004, *z* = 0.42, *p* = 0.67; Zero β = −0.05, *z* = −0.54, *p* = 0.59) nor mean electro-dermal (Count β = −0.59, *z* = −0.79, *p* = 0.43; Zero β = −18.21, *z* = −0.63, *p* = 0.53) arousal predicted like counts or excess zeros. Therefore, we retained a simpler NB model for inference.

The null effect in H3a raises the question whether post type moderates the affect-action link. Authentic, non-sponsored content may allow affective resonance to manifest behaviorally, whereas persuasive intent may attenuate that pathway. Hypothesis H3b proposes that this relationship is stronger for organic posts than for sponsored posts. To test H3b, we fitted a two-level logistic mixed-effects model predicting the probability that a user “likes” a given post (Liked_Any_Post = 1 vs. 0). Posts (Level 1) were nested within participants (Level 2, RespID as the participant). Fixed-effects predictors included sponsor status (0 = organic, 1 = sponsored), person-mean-centered facial expression arousal (FEA_Positive_C), and person-mean-centered electrodermal arousal (GSR_Frequency_C). The model also included two cross-level interactions: sponsor × FEA_Positive_C and Sponsor × GSR_Frequency_C. A random intercept for RespID (variance components) accounted for between-person heterogeneity. Estimation used Fisher’s penalized quasi-likelihood with 100 iterations and a convergence criterion of 1 × 10^6^.

Random intercept variance was σ0^2^ = 27.12 (SE = 5.95, *p* < 0.001), yielding an ICC of 27.12/(27.12 + π^2^/3) ≈0.84. Model fit was acceptable (−2 log L = 12 450.3; AIC = 12 464.3). Sponsored posts were significantly less likely to be liked than organic posts (OR = 0.36, *p* = 0.004); however, neither within-person deviations in facial-expression arousal (FEA_Positive_C) nor electrodermal arousal (GSR_Frequency_C) predicted liking (“main effects”), and neither interaction with sponsor type reached significance (all *p* > 0.45) (Table 6). Random intercept variance remained large (ICC ≈0.84), indicating that most variability in “liking” lies between individuals, not across posts. We found no evidence that momentary, within-person fluctuations in either facial or electrodermal arousal differentially drive the liking for sponsored vs. organic content. Therefore, Hypothesis 3b is not supported.

**TABLE 6:** Random-intercept logistic regression predicting post-level “like” (H3b).

Effect	*b*	SE	OR	95% CI for OR	*z*	*P*
**Fixed effects**						
Intercept (organic posts)	−0.85	0.20	0.43	[0.32, 0.57]	−4.25	<0.001
Sponsor (vs. organic)	−1.02	0.35	0.36	[0.20, 0.63]	−2.90	0.004
FEA_Positive_C	0.01	0.02	1.01	[0.97, 1.05]	0.50	0.62
GSR_Frequency_C	0.00	0.10	1.00	[0.82, 1.22]	0.05	0.96
Sponsor × FEA_Positive_C	0.02	0.03	1.02	[0.97, 1.07]	0.76	0.45
Sponsor × GSR_Frequency _C	0.10	0.20	1.11	[0.73, 1.68]	0.50	0.62

CI = Confidence Interval; GSR = Galvanic Skin Response; FEA = Facial Expression Analysis; SE = Standard Error; OR = Odds Ratio.

## 4 Discussion

This study conceptualized social media browsing as a micro-customer journey–an immersive, temporally granular experience shaped by ongoing affective fluctuations. Rather than simply extending traditional customer journey models (Lemon and Verhoef, 2016), our findings challenge the core assumption embedded in these models: emotional engagement across the journey can be linearly tracked and leveraged for persuasive design. The emotional rhythm we observed, characterized by a neutral baseline punctuated by unpredictable affective spikes (Layer 1), suggests that emotional continuity may not exist at the granularity where decisions are made.

Layer 1 confirmed that the baseline experience of browsing was characterized by a predominantly neutral emotional tone, occasionally punctuated by peaks in arousal and positive affect. Affective findings reinforce the notion that scrolling is a cognitively active, but emotionally restrained activity (Ochsner et al., 2002). The one pronounced moment of shared emotional elevation (indicated by the positive affect) aligns with the notion of “affective saturation” in digital environments, where marked positive spikes in user engagement or emotion may stand out against a backdrop of general neutrality (Gennaro et al., 2021; Mulyono and Saskia, 2021). This undermines hierarchical stage-based interpretations of consumer decision-making and highlights the dynamic and recursive nature of affective states in real-time interactions. Rather than progressing smoothly from awareness to consideration to action, the microjourney is shaped by attentional shifts, transient salience, and intermittent disruptions–many of which do not cohere into stable narratives or decision outcomes.

This perspective nuances the S–O–R and CAB models by positioning the “Organism” not as a passive mediator but as an active site of perceptual and affective filtering. It is not merely the type of stimulus that determines behavior, but also the timing within the affective rhythm, its congruence with emotional expectations, and its ability to cut through a stream marked by cognitive habituation. Our findings point out the need for more granular, time-sensitive revisions to consumer journey frameworks that acknowledge the volatility and context dependence of emotional micromoments. This foundational affective rhythm (Layer 1) is crucial because it creates the conditions under which subsequent disruptions (Layer 2) and behavioral translations (Layer 3) are interpreted.

Layer 2 revealed that sponsored content does not significantly elevate arousal but does meaningfully reduce affective neutrality. Although not statistically significant at conventional alpha thresholds, this trend is conceptually meaningful. It resonates with studies suggesting that organic posts, often comprising social or entertaining content from known peers or influencers, elicit “authentic” positive affect (Kim and Kim, 2020; Reade, 2021; Wang and Weng, 2024). By contrast, sponsored content may evoke ambivalence because of perceived persuasive intent or emotional incongruence (Zimand Sheiner et al., 2021), thereby dampening overt positivity. This pattern suggests that emotional gratification on platforms such as Instagram may be contingent on content origin, reinforcing the idea that digital environments are affectively stratified not only by content, but also by their social-symbolic role (Saleem and Iglesias, 2020). These findings underscore the importance of message authenticity and audience-content congruence in shaping micro-level affective responses, although statistical confirmation remains marginal. The lack of differential negativity suggests that sponsored content is not actively disliked, but may instead fail to elicit engagement, a subtler form of emotional disengagement. Rather than provoking aversion, it may be merely less engaging, which is arguably a more subtle and insidious effect. This aligns with an “emotional disengagement hypothesis,” where persuasive content is tolerated but not embraced, dampening both peaks and valleys of emotional resonance. Organic posts elicited substantially more neutral frames than sponsored posts. At first glance, this may appear paradoxical as to why content without explicit emotional cues dominates neutral responses in organic posts, a pattern that suggests that cognitive engagement may occur independent of overt affect. Crucially, neutral facial expressions in this context should not be misinterpreted as the absence of emotions. Instead, they may signify affective ambiguity, cognitive processing, or low-arousal attention. Such responses may reflect a “scrolling mode.” This state is characterized by sustained attentional but flattened affective engagement (Meier et al., 2023; Piya and Adhikari, 2023). The reduction in neutral frames during sponsored content suggests that such posts interrupt this mode, potentially triggering more emotionally polarized reactions, although not significantly positive or negative. This aligns with the “affective volatility” in digital environments, where interruptions by salient or incongruent stimuli (e.g., advertisements) trigger state shifts without necessarily increasing valence extremes (Gaspelin et al., 2012). Thus, sponsored posts may not be more emotional but less neutral, hinting at a disruption of affective flow and possibly cognitive reappraisal.

From a psychophysiological perspective, this suggests higher variability in stimulus salience among organic content, possibly due to social, contextual, or multimodal features (Yi et al., 2025). The lack of significance may be due to individual differences in tonic arousal or habituation effects within browsing streams. Although the frequency of activation may vary marginally by content type, the intensity of these arousal spikes remains largely stable. This reinforces the idea that post type modulates attention rather than emotional intensity. These findings align with theories positing that arousal in social media is a function of novelty detection rather than emotional valence (Clerke and Heerey, 2023; Prowten et al., 2024) and sponsored posts, while salient regarding format or targeting, do not automatically generate stronger physiological engagement.

Collectively, the results reveal a nuanced picture: sponsored posts do not overtly increase either positive or negative affective load, nor do they evoke stronger physiological arousal. Instead, they reduce affective ambiguity; that is, they are less likely to elicit neutral, cognitively curious expressions. This suggests that sponsored content subtly displaces users from the habitual, semi-engaged “scrolling mode” by injecting content that is perceptually dissonant or cognitively taxing. This interpretation aligns with the recent studies on affective design and digital interruption science. Organic content may act as an affective “background hum” sustaining platform engagement, whereas sponsored posts create micro-disruptions that prompt cognitive (but not necessarily emotional) re-evaluation. Such disruptions may not immediately register as affective valence shifts but may accumulate downstream cognitive load or resistance (Hsu et al., 2021).

Critically, this analysis challenges simplistic assumptions about the emotional power of persuasion: Commercial content may not need to arouse more to affect behavior; it merely needs to subtly disrupt affective flow. Thus, the absence of overt affective divergence may, paradoxically, indicate a deeper mechanism of engagement modulation. These findings contribute to the emerging models of affective design, algorithmic emotional curation, and attentional fragmentation in social media environments. They further suggest that the affective architecture of digital feeds is subtly but powerfully stratified, not necessarily by what evokes emotion but what interrupts or sustains emotional flow.

Although marginal effects were observed for positive affect, the most consistent finding was that advertisements interrupted the scrolling flow by decreasing emotional ambiguity. This supports our predictive coding-based interpretation that users form implicit expectations of emotional continuity in the feed and sponsored posts violate these predictions, prompting reappraisal. However, the absence of increased arousal or consistent positivity suggests dissociation between attentional capture and motivational engagement (Breuer et al., 2021). Though users may cognitively register the disruption caused by a sponsored post, this disruption lacks the affective valence or goal congruence necessary to trigger an intentional behavioral response (Imbir et al., 2023). This aligns with studies differentiating bottom-up attentional salience from top-down motivational relevance (Sutherland et al., 2017; Duncan et al., 2023). Consequently, digital environments marked by high variability in affective tone and commercial interruptions may foster a context in which attentional engagement is frequent but motivational engagement is rare. This insight bridges consumer neuroscience with emerging debates in affective computing, suggesting that systems designed to trigger interaction must consider not only emotional arousal but also its direction, interpretability, and alignment with user goals. Contrary to early persuasion models suggesting that emotional intensity drives behavioral responses (Petty and Cacioppo, 1986), our results imply a subtle affective mechanism. Sponsored posts do not generate a strong affect; rather, they interrupt it. This aligns with emerging literature on “cognitive friction” in digital persuasion (Ericson, 2022) and underscores the need to distinguish between emotional charge and emotional disruption in affective computing and digital marketing research. These findings refine our understanding of the “Organism” component in the S–O–R model – the internal state is not merely reactive but shaped by continuous affective context. Disruptions may signal salience; however, without valence clarity or motivational relevance, they may fail to activate the response component.

Layer 3 found no significant predictive relationship between biometric arousal or valence and liking behaviors. The absence of a significant link between momentary affective arousal/valence and engagement behavior (as formulated in RP3a and RP3b) invites several, non-mutually-exclusive explanations. One concerns the performative and normative functions of “likes” on social media: rather than reflecting an internal affective reaction to the content, likes can serve to maintain social reciprocity, signal group affiliation, or adhere to platform norms, thereby weakening their direct connection to emotional states. Another concerns the role of habitual, low-effort digital behaviors. In fast-paced, scroll-based environments, liking can become an automatic, cue-driven micro-interaction, initiated with minimal cognitive appraisal and decoupled from transient physiological arousal or valence shifts. From a methodological standpoint, our biometric measures capture fine-grained, short-lived changes in sympathetic activation and facial expressions, which may not temporally align with the more socially and habitually mediated act of liking. Moreover, the constrained operationalization of engagement–chosen to preserve biometric signal quality–reduced behavioral variability, potentially lowering statistical power to detect subtle affect–behavior associations. Theoretically, these findings challenge the foundational assumption in CAB and S–O–R models that affective states translate directly and reliably into observable consumer behavior. In low effort, habitual contexts, such as feed browsing and momentary affective changes, may lack the motivational force or decision relevance to influence engagement. Our data suggest that the link between emotion and behavior is not merely a matter of emotional intensity but of functional alignment: for an affective response to manifest as an action, it must coincide with motivational salience, attentional readiness, and situational affordances that support behavioral expression. In other words, affect may be necessary, but not sufficient. This finding aligns with dual-process theories (Kahneman, 2011), which posit that behavioral outputs in digital environments often emerge from System 1 heuristics or habitual scripts rather than affect-driven deliberation.

These insights offer a transdisciplinary contribution to both affective computing and marketing strategies. For marketers and platform designers, our results are cautious against the assumption that emotional disruption is equivalent to persuasion. Advertisements that disrupt emotional flow may draw attention, but do not necessarily translate into engagement. Interestingly, the most robust finding was not a surge in negative or positive emotional expressions, but a significant reduction in neutral frames during sponsored posts. This suggests that native ads subtly shift users out of an emotionally ambiguous or inattentive state without provoking backlash or triggering overt emotional responses. For practitioners, this implies a strategic advantage: users are not overtly rejecting integrated advertising, nor is their browsing experience markedly disrupted. Thus, the debate is not whether advertising should appear in these spaces, but how to design it to harmonize with the scrolling flow of users.

In the broader context of customer journey mapping, these findings position native advertisements not as interruptions but as low-resistance brand touchpoints. Their neutrality, interpreted not as ineffectiveness but as a cognitive presence without affective conflict, may offer a valuable form of microexposure in the awareness or consideration stages, particularly when trust and familiarity are cultivated over time.

This study had some limitations that temper its conclusions. While the simulated feed, was deliberately designed to emulate the mobile form factor, content ratio, and self-paced scrolling dynamics of real-world platforms, it necessarily lacked certain features, such as inclusion of algorithmic personalization, peer comments or dynamic ad targeting, that may influence affective and behavioral responses. Such elements are known to enhance engagement and emotional intensity through mechanisms such as relevance-matching and social proof; their absence in our design may have attenuated overall response magnitudes and narrowed interindividual variability. The substantial between-subject variance observed in our data (e.g., ICC values) suggests that stable trait-level factors, such as attitudes toward advertising, familiarity with the platform, or personality dispositions, may moderate responses to both sponsored and organic content. These individual differences were not assessed here but represent important avenues for future research, particularly in designs that combine the experimental control of standardized stimuli with participant-level tailoring and trait measurement.

A further limitation is the absence of any subjective self-reports of emotional experience. Although the GSR and automated FEA capture physiological proxies of arousal and valence, they cannot fully encompass cognitive appraisal or conscious emotional states. Without self-reported measures, it is not possible to assess the degree of alignment between physiological responses and participants’ perceived emotions. Future research should consider triangulation via post-exposure surveys, interviews, or real-time experience sampling methods to provide a more comprehensive account of digital content engagement.

In addition, the study did not include an explicit manipulation check for advertisement recognition. While all sponsored posts carried the mandated “Sponsored” disclosure, we cannot rule out the possibility that some participants did not consciously register this label. This omission introduces interpretive ambiguity. For example, an emotionally neutral response to a sponsored post may reflect non-recognition rather than genuine disengagement. Including a brief recognition check in future work would help clarify the role of conscious ad perception in shaping affective and behavioral responses. In line with this, future studies could also adopt a multi-method approach that tracks whether and when users attend to the source of a post (e.g., via eye-tracking) and pairs this with retrospective think-aloud interviews probing whether likes reflect genuine content appreciation or social/normative motivations. In particular in emerging areas of algorithmic influence and micro-interactions, those designs would help disentangle the habitual and performative components of engagement from those driven by affective arousal or valence. Finally, future research could embed personalization features within controlled paradigms, to capture the full complexity of emotional, cognitive, and social processes in real-world social media environments.

In conclusion, this study bridges the mechanistic understanding of affective processing in decision neuroscience and its strategic application in marketing science by translating biometric insights, specifically electrodermal and facial-affective responses, into actionable knowledge about customer experience, platform design, and native advertising strategy. By situating transient affective states within the early stages of the customer journey framework, this study offers two contributions: (1) enhancing psychological models of online decision-making processes and (2) informing emotionally congruent marketing designs in algorithmically curated environments. Although momentary emotional shifts shape the subjective browsing experience, they do not automatically translate into behavior. Therefore, emotional design in digital environments must move beyond arousal metrics and attend to the subtleties of rhythm, congruence, and context. Thus, both theory and practice can fully account for how digital content navigates and occasionally reshapes the emotional landscape of everyday life.

## Data Availability

The datasets presented in this article are not readily available because they are the basis for ongoing research. Data may be available from the corresponding author on reasonable request after completion of the overarching research project (planned end October 2026). Requests to access the datasets should be directed to maike.huebner@hs-ruhrwest.de.
